# Chronic Non-Suppurative Otitis Media*A paper read before the American Laryngological, Rhinological and Otological Society, Washington, D. C., May, 1897.

**Published:** 1897-11

**Authors:** S. Mac Cuen Smith

**Affiliations:** Philadelphia, Pa., Clinical Professor of Otology, Jefferson Medical College; Surgeon in Charge Ear, Throat and Nose Department, Germantown Hospital


### CHRONIC NON-SUPPURATIVE OTITIS MEDIA.*

BY S. MAC CUEN SMITH, M.D., PHILADELPHIA, PA.,

Clinical Professor of Otology, Jefferson Medical College; Surgeon in Charge Ear, Throat and Nose Department, Germantown Hospital.

My object in presenting a paper on this subject is not to offer anything especially new in therapeutic measures, but with the hope that it will create sufficient interest to stimulate an active and exhaustive discussion of this most intractable disease, which would no doubt prove of interest and benefit to us all.

Being pre-eminently an affection that depends for its existence upon some abnormal condition ot the nose and throat, we must necessarily consider the naso-pharynx not only as the most important etiologic factor, but also a matter for sober thought when the more serious subjects of treatment and prognosis are under consideration. This becomes all the more important from the fact that the disease (in its early stages) is one that is practically without symptoms. In other words, the pathologic changes in the naso-pharynx and tympanum will have continued for some time before the aural implication becomes manifest; furthermore, the function of the conducting apparatus will often become seriously impaired before the patient realizes that his ears are sufficiently at fault to require treatment. It is generally at this stage, or even when the disease *is more advanced*, that the patient applies for relief, suggesting that he is only suffering from one of the many "colds in the head."

It is quite probable that suppurative diseases of the ear constitute the greatest number of all aural troubles, and, furthermore, occasion most of the serious complications found in otology; but we feel justified in claiming that few diseases in the wide domain of medicine are capable of perpetuating an existence so absolutely miserable and distressed as afflicts one suffering from the advanced stages of the disease under consideration. It is for the alleviation and cure of these unfortunates that our unceasing efforts should be

*A paper read before the American Laryngological, Rhinological and Otological Society, Washington, D. C., May, 1897.

earnestly applied, hoping that we may be able to continue, or even excel, the progress shown in the past few years.

Several groups or varieties of cases might be considered, each having more or less distinctive features, but for clinical purposes it will be sufficiently accurate to regard these several forms as simply successive stages of the same progressive disease, beginning with the simple catarrhal, and ending with the sclerotic, or even the more advanced atrophic changes, involving finally the internal ear itself, and accompanied by all the discomfort of distressing deafness, tinnitus, vertigo, etc.

In support of the claim that all chronic non-suppurative inflammations of the tympanum are primarily caused by an extension of some existing abnormal condition of the naso-pharynx, we need but call attention to the well-known fact that all manner and condition of people are effected to the same extent. Those surrounded by the best possible circumstances to promote and preserve perfect health, are afflicted in precisely the same manner as those occupying the humbler walks of life, and therefore not in a position to enjoy the benefits of modern sanitary and hygienic living. We do not believe, therefore, that the relative general health of individuals (except those influenced by syphilis and allied diseases), has any material influence upon the development of the disease under consideration. The true primary exciting cause must be looked for in the form of one of the common catarrhal conditions of the naso-pharynx, and, therefore, very few patients materially improve without energetic treatment of these organs.

Further consideration of this subject will be confined to that advanced form of disease characterized by more or less obstruction of the Eustachian tube (a complete obliteration of their lumen being infrequent), marked retraction of the membrana tympani, with a greater or less degree of thickening and opacity; depression or displacement of the ossicles, with subsequent complete ankylosis of their articular surfaces, and finally, the membrane and ossicles bound down by firm bands of adhesions to the promontory, thus suspending their respective functions, and causing a complete obstruction to the transmission of sound. If in addition we have impaction (even bony union) of the stapes into the fenestra ovalis, and subsequent involvement of the labyrinth or auditory nerve, is it to be wondered that a most miserable existence is the result, or that so many surgical and other measures, of doubtful utility, have been from time to time employed for their relief?

Although great progress has been made in aural therapeutics during the past few years, much, nevertheless, remains to be accomplished in the pathology and treatment of this troublesome disease. Manifestly the essential point in treatment is to correct or remove the cause; treatment applied to the ear alone can only be successful when the disease is of recent date, and *Nature* unaided improves the exciting cause in the naso-pharynx. Not only must the exciting catarrhal condition of the nose and throat be improved as far as possible, but we deem it of the greatest importance that free *nasal* respiration shall be established, as it is only by this correct respiratory action that proper ventilation of the tympanic cavity can be obtained. Cases with little or no involvement of the internal ear can usually be benefited to a greater or less degree. If there is but little change in the texture or position of the membrana tympani, no rigidity of the ossicles, and an absence of atrophic changes, involving the tympanic cavity, very little and simple treatment will frequently produce satisfactory results. On the other hand, a long and vigorous treatment is usually required, when we attempt the relief of patients in whom the drum-head has undergone various changes, and is firmly attached by bands of adhesion to the tympanic walls, and associated with ankylosis and a malposition of the ossicles.

It would seem folly to hope for relief in cases of this kind, without first causing liberation of the membrana tympani and ossicles. In recent years our method of treatment has been to make an incision through the membrana tympani, either anterior or posterior (one or both) to the long handle of the malleus, and extending along its entire length. Through these openings is introduced a small knife (the cutting edge being at right angle to the handle), with which we carefully divide the bands of adhesions; then with a small hook we grasp the handle of the malleus high up, and with gentle but firm traction endeavor to break up the ankylosis. A few drops of sterilized oil (liquid albolene) are now introduced into the tympanic cavity, the object being to supply a non-irritating lubricant, which will both facilitate the natural movements of the drum-head and ossicles, and also assist in preventing future adhesions. The camphor-menthol solution, as suggested by Dr. Bishop, can be substituted for the plain oil, when a mild, stimulating application is indicated. The canal is now well filled with iodoform gauze (care being taken not to impinge upon the membrana tympani), and the patient is required to remain in bed about forty-eight hours. The gauze should be removed on the second or third day, at which time the incised drum will have become wholly reunited.

Careful massage of the membrana tympani and ossicles with Siegle's pneumatic speculum is now performed, and should be repeated at intervals of three or four days, until a satisfactory position is permanently established. In this connection it may be of interest to state that the experience of the writer has thoroughly convinced him that we can obtain much better results from massage by the use of Siegle's pneumatic speculum than by the employment of the various elaborate and costly appliances generally used for this purpose.

When necessary, additional injections of oil can be made, by the aid of a long hypodermic needle, directly through the drum-head, or by way of the Eustachian tube. With a view of still further promoting absorption, and for its general alterative effects, the patient is given from ten to twenty hypodermic injections of hydrochlorate of pilocarpin, at intervals of two or three days. Internally we administer $\frac{1}{6}$ grain each of iodine and bromine, 1-100 grain of phosphorus, and 1-40 grain of strychnia, to be taken four times daily for several weeks. It is assumed that the naso-pharynx (including the Eustachian tube) will have received proper care before the above line of treatment is instituted. The result of this treatment has been very satisfactory in many cases, relieving the tinnitus and vertigo, and materially improving the hearing; furthermore, beneficial results have been obtained after other methods of treatment failed to bring about improvement.

During a period of several years we have conducted a large series of experimental operations for the relief and cure of the disease under consideration, and as a result of these observations we may conclude: (1) that the best and most permanent results will be obtained from conservative treatment, as outlined above; (2) that, as the operative measure herein suggested is practically without risk, its adoption seems advisable before more formidable operations are resorted to; (3) that the majority of cases without labyrinthean implication will be relieved of tinnitus and vertigo and greatly improved in their hearing power; (4) that chronic cases with internal ear involvement do not offer hope for a continued improvement in hearing power, but may frequently be relieved of tinnitus and vertigo; (5) that *excision* of any part of the conducting apparatus is only justifiable when relief from severe tinnitus and vertigo is the object of treatment, and other measures have failed.

